# Template-free Synthesis of One-dimensional Cobalt Nanostructures by Hydrazine Reduction Route

**DOI:** 10.1007/s11671-010-9807-7

**Published:** 2010-10-01

**Authors:** Liying Zhang, Tianmin Lan, Jian Wang, Liangmin Wei, Zhi Yang, Yafei Zhang

**Affiliations:** 1 National Key Laboratory of Nano/Micro Fabrication Technology, Key Laboratory for Thin Film and Microfabrication of the Ministry of Education, Institute of Micro and Nano Science and Technology, Shanghai Jiao Tong University, 200240, Shanghai, China; 2 School of Materials Science and Engineering, Shanghai Jiao Tong University, 200240, Shanghai, China

**Keywords:** Cobalt nanowires, Magnetic field assistance, Hydration hydrazine reduction

## Abstract

One-dimensional cobalt nanostructures with large aspect ratio up to 450 have been prepared via a template-free hydrazine reduction route under external magnetic field assistance. The morphology and properties of cobalt nanostructures were characterized by scanning electron microscopy, X-ray diffractometer, and vibrating sample magnetometer. The roles of the reaction conditions such as temperature, concentration, and pH value on morphology and magnetic properties of fabricated Co nanostructures were investigated. This work presents a simple, low-cost, environment-friendly, and large-scale production approach to fabricate one-dimensional magnetic Co materials. The resulting materials may have potential applications in nanodevice, catalytic agent, and magnetic recording.

## Introduction

In recent years, nanostructure materials have been actively studied due to their novel properties and potential applications. Among them, much attention has been focused on one-dimensional (1D) magnetic materials such as Fe, Co, and Ni due to their potential applications in nanodevice, biosensor, and magnetic recording [1-3]. 1D cobalt nanostructures with uniform shape and high purity have become increasingly required for specific uses in many areas, such as in high-density information storage, magnetic sensors, commercial batteries, and catalysts.

Various approaches have been developed to synthesize cobalt nanostructures [4-6]. For example, Puntes et al. prepared Co nanodisks by rapid decomposition of carbonyl cobalt in the presence of trioctylphosphane (TOP) and oleic acid (OA) [7]. Legrand et al. applied a physical method to synthesize 3D supra-organization of Co nanocrystals [8]. The most common method to fabricate 1D Co nanostructures is based on porous anodic aluminum oxide (AAO) templates. Li et al. prepared Co nanowire arrays in alumina arrays by using a chemical electrodeposition method [9]. Other templates such as polyaniline or polycarbonate membranes, diblock copolymer, and mesoporous silica have also been applied to fabricate magnetic Co nanowires [10-12]. In view of the complexity of multi-step template preparation and low production, it is imperative to develop simpler template-free methods for the fabrication of magnetic Co nanowires.

In recent years, our research group has reported a chemical solution reduction approach for fabricating 1D Ni nanostructures assisted by magnetic fields [13-15]. This may be a more promising method to prepare cobalt nanostructures in terms of its low cost and potential for large-scale production. In this study, we fabricated Co nanowires with large aspect ratio under normal pressure in absence of any templates or surfactants. The influence of reaction temperature, Co ion concentration and magnetic field intensity on the formation and morphology of Co nanowires were investigated. The magnetic properties of Co nanostructures with different morphology were also evaluated.

## Experimental

All chemicals were of analytical grade without further purification. In a typical synthesis, an appropriate amount of CoCl_2_·6H_2_O was dispersed in 50 ml of ethylene glycol in a 250-ml beaker, where the concentration of CoCl_2_·6H_2_O varied from 0.01 to 0.1 M. An appropriate amount of 85 wt% N_2_H_4_·H_2_O solution and 5 M NaOH solution was then added to the mixed solution with constant stirring. An NdFeB permanent magnet was placed beneath the beaker to apply an external magnetic field to the reaction system. The magnetic field intensity on the inner surface of the beaker was controlled from 0.005 to 0.40 T at room temperature by adjusting the distance between the beaker and the magnet, and the intensity of the magnetic field was measured by using a Tesla meter. The final mixture was then allowed to react at temperature of 40, 60, and 80°C for 30 min. The chemical reaction for the synthesis of the Co nanowires can be expressed as below:

$$2{\text{Co}}^{2+}+{\text{N}}_{2}{\text{H}}_{4}+4{\text{OH}}^{-}=2\text{Co}↓+\;{\text{N}}_{2}↑+\;4{\text{H}}_{2}\text{O}$$

After the beaker cooled down to room temperature, the gray solid product floating on the beaker was collected by using a magnet. Then it was washed with distilled water and absolute ethanol several times and finally dried in a vacuum oven at 60°C for 24 h.

The size and morphology analyses were performed using a field emission scanning electron microscope (SEM, Ultra55, Zeiss). The crystal structure was characterized by an X-ray polycrystalline diffractometer (XRD, D8 Advance, Bruker) using Cu Ka radiation (λ = 1.54056 nm) with graphite monochromator. The hysteresis properties were measured on a vibration sample magnetometer (VSM, Lake Shore 7400).

## Result and Discussion

**Figure 1:**
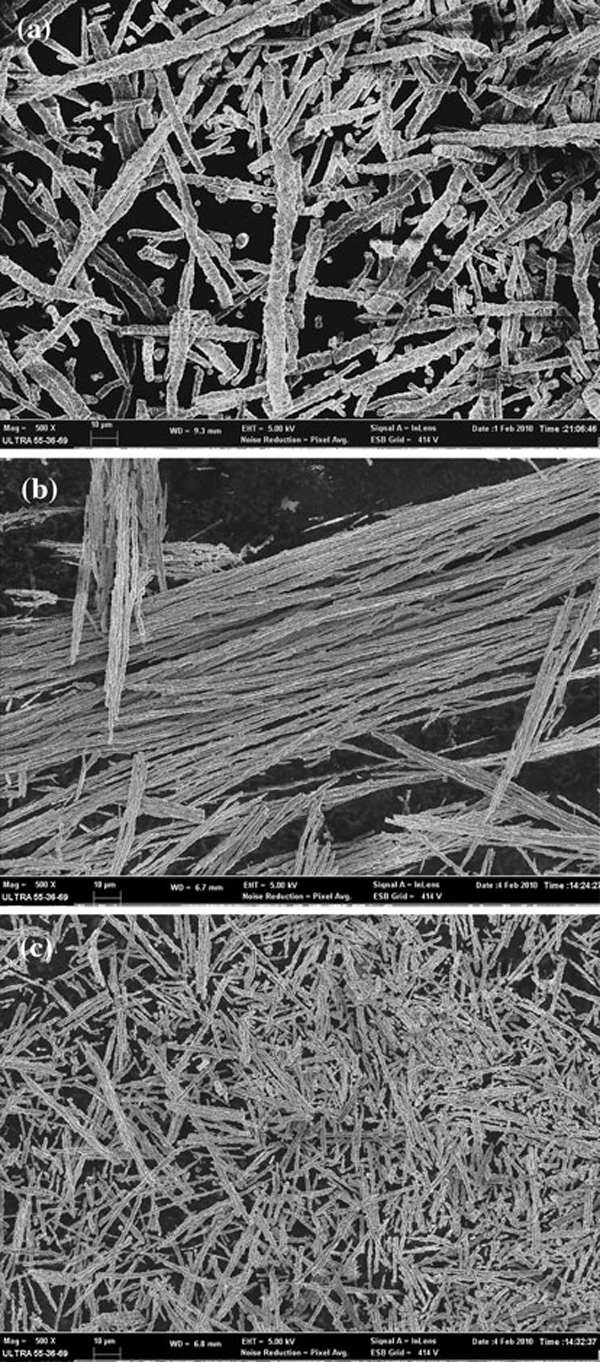
**SEM images with the same magnification times of Co nanostructures prepared at different reaction temperatures: a 40°C; b 60°C; c 80°C**.

Figure 1 shows the SEM images of resulting products prepared under different reaction temperatures of 40, 60, and 80°C, in which Co ion concentration was 0.01 M and the magnetic field of 0.4 T. It is apparent that the mean diameter of the wires prepared at higher temperature is much less than at lower temperature, whereas the aspect ratio of the wires increased with the temperature firstly and reached its highest value of about 450 at 60°C and then decreased with increased temperature. The diameter is about 4 μm at 40°C (see Figure 1a) and 800 nm at 60 and 80°C (see Figure 1b, c), respectively. The wires fabricated at 60°C are much smoother, longer, and more uniform. This may be derived from the thermodynamic influence on the nucleation velocity and the subsequent fast growth of the crystals, which results in smaller size of the Co particles [16,17]. When the reaction temperature is sufficiently high up to 80°C, the average length of the corresponding Co nanowires appears to decrease from 350 to 200 μm as a result of the influence of thermal kinetics interaction. Consequently, we choose temperature of 60°C as the optimal reaction temperature for the preparation of Co wires.

**Figure 2:**
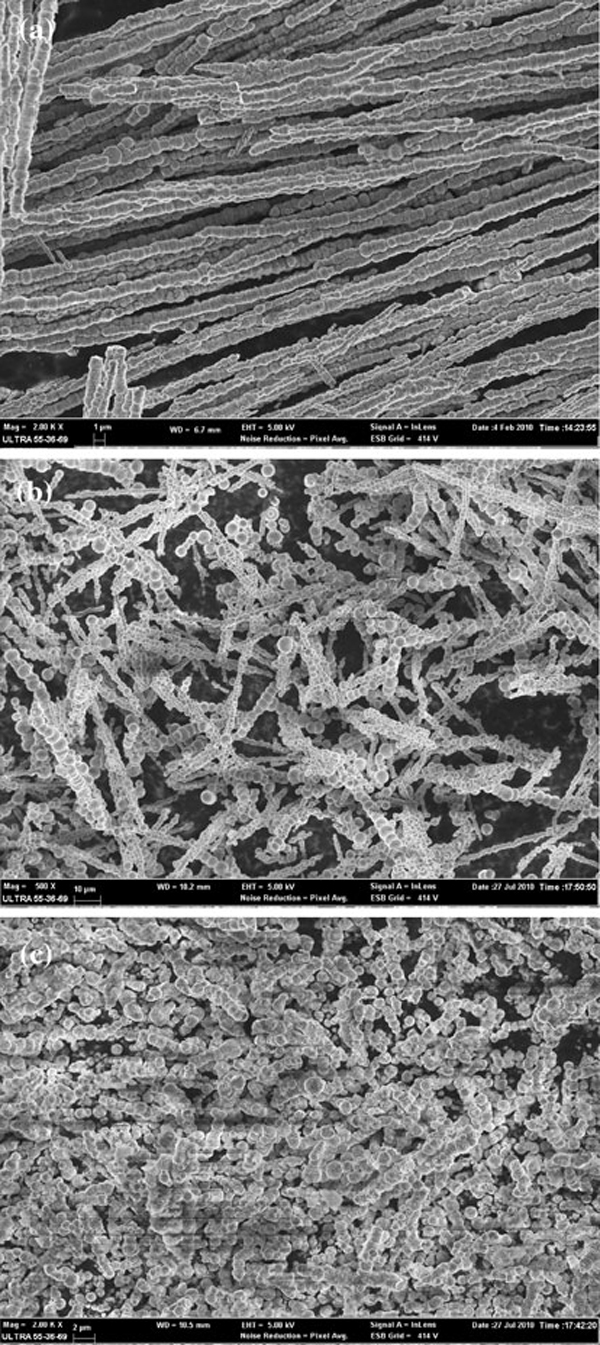
**SEM images of products prepared in different Co ion concentrations under a 0.40 T magnetic field: a 0.01 mol/l; b 0.1 mol/l; c 0.5 mol/l**.

The influence of Co ion concentration on morphology of Co wires has also been investigated. Figure 2 shows the SEM images of products with different Co ion concentration under the same external magnetic field of 0.4 T. It can be seen that the Co ion concentration has a strong influence on the morphology of products. The average diameter of the wires increases with increased Co ions concentration. It is about 800 nm for concentration of 0.01 M (see Figure 2a) and about 1.5 μm for 0.1 M (see Figure 2b), whereas the average length decreases from 350 to 50 μm. When the concentration reached 0.5 M, the corresponding products tend to further agglomerate and grow thicker, which results in the potato-like structure with the average diameter of 2 μm (see Figure 2c). The possible reason is that the primary Co crystallites tend to aggregate together to form spherical particles to decrease the surface energy when the concentration increased.

**Figure 3:**
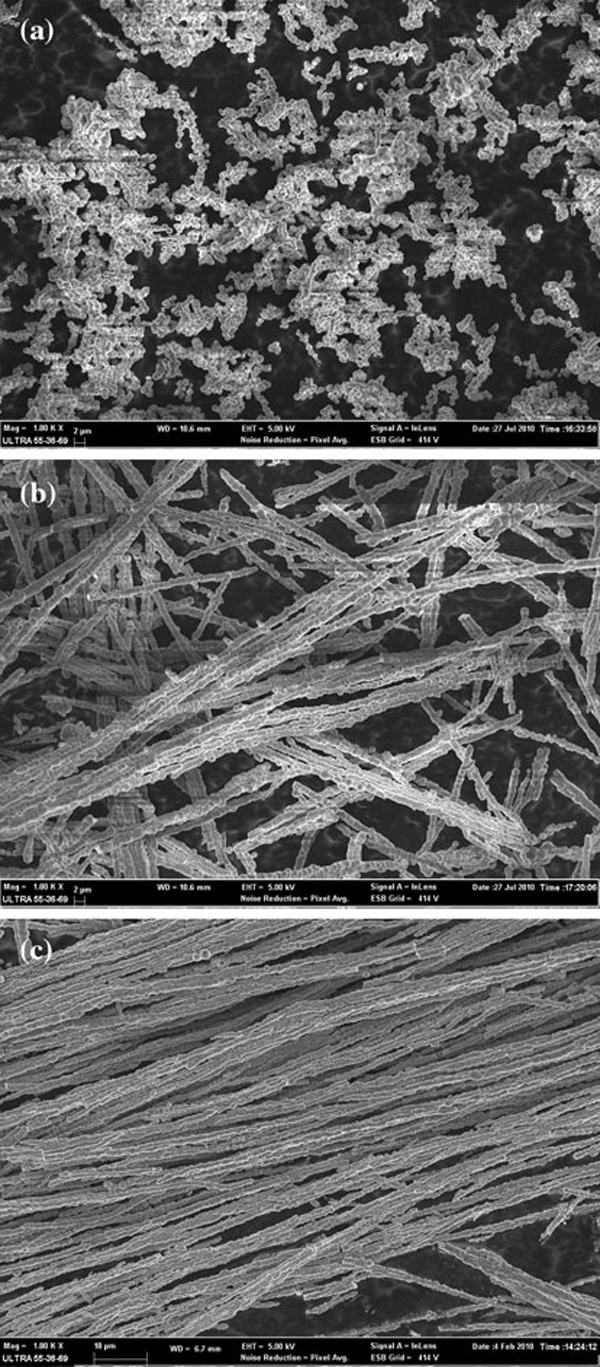
**SEM images of samples prepared under different magnetic fields: a 0 T; b 0.15 T; c 0.40 T**.

In order to investigate the effect of the external magnetic field on the morphology of the resulting products, magnetic fields with different intensity were applied, where the Co ion concentration and the reaction temperature were 0.01 M and 60°C, respectively. As shown in Figure 3a, only some bulky particles were observed in the absence of the external magnetic field. Under a low magnetic field of 0.15 T, some short, unsmooth, and thick wires were obtained. The average length and diameter are respectively about 200 and 1.5 μm (see Figure 3b). The surface of the wires was irregular, at which lots of Co particles are not aligned along its magnetic anisotropy direction in order to reduce the surface energy. When the intensity of the external magnetic field was increased to 0.40 T, the corresponding Co nanowires appeared to be significantly elongated. The orientation of the Co nanoparticles is further promoted, which results in some parallel self-assembled arrays of Co nanowires. The wires have aspect ratio of about 450 with average diameter of 800 nm and length up to 350 μm (see Figure 3c). It can be concluded that the magnetic field had played a very important role in forming 1D nanostructure, and therefore the length of the wires can be easily controlled by adjusting the intensity of the external magnetic field.

**Figure 4:**
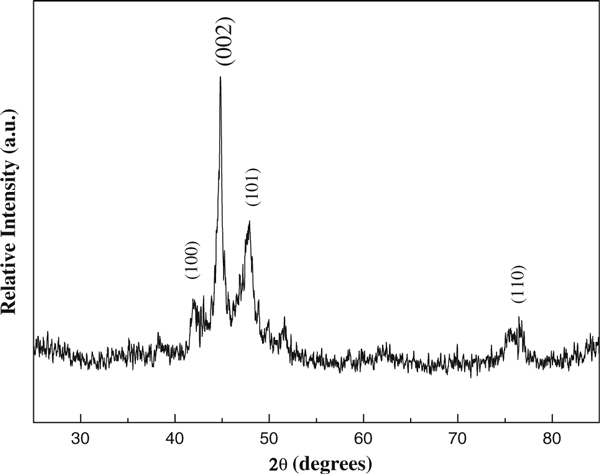
**XRD pattern of Co nanowires prepared at 60°C under a 0.40 T magnetic field**.

The XRD pattern of the Co nanowires prepared at 0.01 M concentration under an external magnetic field of 0.40 T is shown in Figure 4. All the diffraction peaks can be well indexed to hexagonal-phase cobalt, with lattice constants of *a* = 2.492 Å and *c* = 4.025 Å, which is well consistent with the standard card (JCPDS 89-4308, P6_3_/*mmc*, *a* = 2.505 Å, *c* = 4.089 Å). Compared with the neighboring peak (101), the relative intensity of peak (002) in patterns increases significantly, which indicates the oriented growth of cobalt crystallites. There is no other impurity observed, suggesting the prepared Co nanowires have high purity.

**Figure 5:**
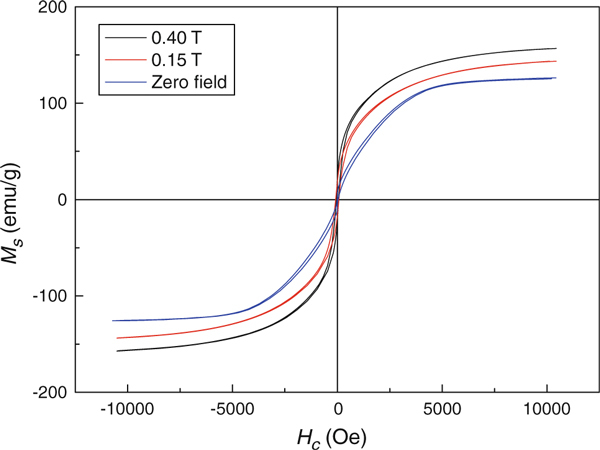
**Hysteresis loops of Co nanostructures measured at different intensity of applied field: Zero-field, 0.15 T-field, and 0.40 T-field**.

Figure 5 displays the hysteresis loops of the samples prepared without an external magnetic field (Zero-field), with a low external magnetic field of 0.15 T (0.15 T-field) and with an external magnetic field of 0.40 T (0.40 T-field), respectively. The saturation magnetization (*M*_*S*_) of the samples is respective 127, 138, and 160 emu/g, which are all smaller than that of bulk Co materials (168 emu/g). The reduced magnetizations result from the surface effect of Co nanostructures, in which lots of Co atoms of the surface are not aligned along its magnetic anisotropy direction in order to reduce the surface energy. Therefore, the magnetic moments of these Co atoms cannot be aligned along the magnetic field due to the strong exchange interaction. On the other hand, the Zero-field sample had coercivity (*H*_*C*_) value of 103 Oe, whereas the 0.15 T-field sample was 84 Oe and 65 Oe for 0.40 T-field one. These values are much lower than that of bulk cobalt material of 1500 Oe. These differences may be attributed to the different magnetic anisotropy manner [18]. The magnetocrystalline anisotropy and shape anisotropy are two main anisotropy energies existed in magnetic materials, which can induce different coercivity. These two anisotropies cause the nanowires to exhibit a vortex-like magnetization distribution so that the moment directions can easily turn parallel to the external magnetic field, which leads to the reduction in the coercivity of the products.

The possible mechanism for the formation of Co nanowires under applied magnetic field may be expressed as following: At first, Co ions were reduced by strong reduction agent of hydrazine hydrate and turned to tiny spherical particles. Then the magnetic Co particles aligned along the magnetic field direction to form one-dimensional nanostructures under the magnetic driving force. The cobalt nanowires retained their linear structure after kept in ultrasonic bath for 10 min, which proved that the nanowires displayed a good mechanical strength.

## Conclusion

A simple, low-cost, environment-friendly approach of preparation magnetic Co nanowires was developed. In this method, the nanowires were fabricated in an ethylene glycol solution at normal pressure by assistant of magnetic field without any templates or surfactants. The prompt wires have average length of up to 350 μm and aspect ratio of up to 450. It was found that the nanowires are elongated with the increasing intensity of magnetic field, and there is no wires formed in absence of magnetic field. The reaction temperature and Co ion concentration have also strong influences on formation and morphology of nanowires. This method provides a new approach to fabricate magnetic nanowires under normal pressure and may be the most promising candidate to produce large-scale magnetic nanowires, which broads their practical applications.

## References

[B1] Puntes VF Krishnan KM Alivisatos AP Science 2001 291 2115 10.1126/science.1057553 11251109 11251109

[B2] Zeng H Skomski R Menon L Liu Y Bandyopadhyay S Sellmyer D Phys Rev B 2002 65 134426 10.1103/PhysRevB.65.134426

[B3] Maaz K Karim S Usman M Mumtaz A Liu J Duan JL Maqbool M Nanoscale Res Lett 2010 5 1111 10.1007/s11671-010-9610-5 20596344 PMC2894180

[B4] Liu XG Wu NQ Wunsch BH Barsotti RJ Stellacci F Small 2006 2 1046 10.1002/smll.200600219 17193167 17193167

[B5] Narayanan TN Shaijumon MM Ajayan PM Anantharaman MR Nanoscale Res Lett 2010 5 164 10.1007/s11671-009-9459-7 PMC2893701 20651915

[B6] Nielsch K Castano FJ Ross CA Krishnan R J Appl Phys 2005 98 034318 10.1063/1.2005384

[B7] Puntes VF Zanchet D Erdonmez CK Alivisatos AP J Am Chem Soc 2002 124 12874 10.1021/ja027262g 12392435 12392435

[B8] Legrand JL Ngo AT Petit C Pileni MP Adv Mater 2001 13 58 10.1002/1521-4095(200101)13:1<58::AID-ADMA58>3.0.CO;2-A

[B9] Li DD Thompson RS Bergmann G Lu JG Adv Mater 2008 20 4575 10.1002/adma.200801455

[B10] Qin J Nogues J Mikhaylova M Roig A Munoz JS Muhammed M Chem Mater 2005 17 1829 10.1021/cm047870q

[B11] Cao HQ Xu Z Sang H Sheng D Tie CY Adv Mater 2001 13 121 10.1002/1521-4095(200101)13:2<121::AID-ADMA121>3.0.CO;2-L

[B12] Ge SH Li C Ma X Li W Xi L Li CX J Appl Phys 2001 90 509 10.1063/1.1327599

[B13] Liu P Li ZJ Yadian BL Zhang YF Mater Lett 2009 63 1650 10.1016/j.matlet.2009.04.031

[B14] Zhang LY Wang J Wei LM Liu P Wei H Zhang YF Nano-Micro Lett 2009 1 49 10.5101/nml.v1i1.p49-52

[B15] Wang J Zhang LY Liu P Lan TM Zhang J Wei LM Kong ES Jiang CH Zhang YF Nano-Micro Lett 2010 2 134 10.5101/nml.v2i2.p134-138

[B16] Peng XG Manna L Yang WD Wiekham J Scher E Kadavanieh A Alivisatos AP Nature 2000 404 5 10.1038/35003535 10716439

[B17] Sun SH Murray CB J Appl Phys 1999 85 4325 10.1063/1.370357

[B18] Kisielewski M Maziewsk A Zablotskii VJ Magn Magn Mater 2005 290-291 776 10.1016/j.jmmm.2004.11.403

